# Wavelet-artificial neural network to predict the acetone sensing by indium oxide/iron oxide nanocomposites

**DOI:** 10.1038/s41598-023-29898-x

**Published:** 2023-03-14

**Authors:** Reza Iranmanesh, Afham Pourahmad, Danial Soltani Shabestani, Seyed Sajjad Jazayeri, Hamed Sadeqi, Javid Akhavan, Abdelouahed Tounsi

**Affiliations:** 1 Faculty of Civil Engineering, K.N. Toosi University of Technology, No. 1346, Vali Asr Street, Mirdamad Intersection, Tehran, Iran; 2 Department of Polymer Engineering, Amirkabir University of Technology, Tehran, 1591634311 Iran; 3 Department of Chemistry, Mashhad Branch, Islamic Azad University, Mashhad, Iran; 4 Department of Chemical Engineering, Abadan Azad University, Khuzestan, Iran; 5 Department of Internet and Wide Network, Iran Industrial Training Center Branch, University of Applied Science and Technology, Tehran, Iran; 6 Mechanical Engineering Department, Stevens Institute of Technology, 1 Castle Point Terrace, Hoboken, NJ 07030 USA; 7 Material and Hydrology Laboratory, Civil Engineering Department, Faculty of Technology, University of Sidi Bel Abbes, Sidi Bel Abbès, Algeria; 8 Department of Civil and Environmental Engineering, King Fahd University of Petroleum & Minerals, Dhahran, 31261 Eastern Province Saudi Arabia

**Keywords:** Chemical engineering, Chemistry

## Abstract

This study applies a hybridized wavelet transform-artificial neural network (WT-ANN) model to simulate the acetone detecting ability of the Indium oxide/Iron oxide (In_2_O_3_/Fe_2_O_3_) nanocomposite sensors. The WT-ANN has been constructed to extract the sensor resistance ratio (SRR) in the air with respect to the acetone from the nanocomposite chemistry, operating temperature, and acetone concentration. The performed sensitivity analyses demonstrate that a single hidden layer WT-ANN with nine nodes is the highest accurate model for automating the acetone-detecting ability of the In_2_O_3_/Fe_2_O_3_ sensors. Furthermore, the genetic algorithm has fine-tuned the shape-related parameters of the B-spline wavelet transfer function. This model accurately predicts the SRR of the 119 nanocomposite sensors with a mean absolute error of 0.7, absolute average relative deviation of 10.12%, root mean squared error of 1.14, and correlation coefficient of 0.95813. The In_2_O_3_-based nanocomposite with a 15 mol percent of Fe_2_O_3_ is the best sensor for detecting acetone at wide temperatures and concentration ranges. This type of reliable estimator is a step toward fully automating the gas-detecting ability of In_2_O_3_/Fe_2_O_3_ nanocomposite sensors.

## Introduction

Atmosphere pollution by volatile organic materials^1^ is a serious problem that threatens environmental protection^2^ and human health^3^. Hence, it is necessary to fabricate reliable sensing devices to make the environment safe and ensure human health^4,5^. So far, different kinds of sensors such as biosensors^6^, electrochemical^7^, and resonant^7^ have been designed for sensing purposes. The resistance-based gas detectors that utilize metal oxide semiconductors for gas sensing have been popular due to their simple operation, high sensitivity, and low cost^8–10^.

The study conducted in 1962 is likely the first evidence that states absorption–desorption of gas on the metal oxide surface changes its conductivity^11^. Changing the solid measurable properties, like its resistance due to the solid–gas interaction is the essence of fabricating the electrochemical gas sensors^12^. Electrochemical sensors have a wide range of applications in the food processing industry^13^, diagnosis of disease^14^, medicine^15,16^, monitoring toxic substances^17^, and detecting explosive residuals^18^ and environmental contaminants^19^.

Acetone is a highly flammable organic compound irritating the skin, throat, nose, and eyes^20,21^.

In recent decades, nanomaterials are used in solar cells^22^, catalytic reactions^23^, heat exchangers^24^, wastewater treatment^25^, energy application^26^, and system sensing^27^. A diversity of mainly nanosized metal oxides, including tungsten oxide^28^, zinc oxide^29^, cobalt oxide^30^, iron oxide^31^, copper oxide^32^, bismuth oxide^33^, samarium oxide^34^, and indium oxide^35^ have already been used to fabricate acetone-detecting sensors. The pure and composite forms of the latest one, i.e., indium oxide (In_2_O_3_), have been suggested as a reliable sensor for detecting gaseous acetone^8,21,36–38^.

Experimental analyses are often expensive, time-consuming, and often contaminated with different levels of uncertainty. Therefore, it is a good idea to develop a model to estimate the acetone-detecting ability of the In_2_O_3_-based sensors. To the best of our knowledge, neither empirical, semi-empirical, nor intelligent methods have been suggested to automate the acetone-detecting ability of the nanocomposite sensors. Consequently, the current research applies a combination of artificial neural networks (ANN) and wavelet transform to fully automate the acetone-detecting ability of the In_2_O_3_/Fe_2_O_3_ nanocomposite sensors. The hybrid wavelet transform-artificial neural network (WT-ANN) utilizes the B-spline wavelet as a transfer function in the hidden layer of the ANN. Since the B-spline wavelet can be readily reshaped, it improves the ANN’s ability to correlate a phenomenon with even a high non-linear nature. Furthermore, the WT-ANN topology has been well-tuned using the genetic algorithm and trial-and-error analysis. This scenario changes the WT-ANN structure and B-Spline shape and monitors the prediction accuracy to find the topology with the highest accuracy. Moreover, this well-tuned WT-ANN helps analyze the effect of nanocomposite chemistry, acetone concentration, and temperature on the sensor performance.

## Experimental data for the acetone sensing by In_2_O_3_/Fe_2_O_3_ nanocomposites

A reliable databank should be available to construct and validate empirical^39^ and semi-empirical^40^ correlations and machine learning methods^41^. Hence, this study has collected 119 datasets about the acetone-detecting ability of the In_2_O_3_/Fe_2_O_3_ nanocomposite sensors from valid references^4,8,9,21^. These laboratory-scale researches have monitored the sensor resistance ratio (SRR) of the In_2_O_3_-based sensors in the air with respect to the acetone as a function of the nanomaterial chemistry, operating temperature, and acetone concentration^4,8,9,21^. The ranges of independent (Fe_2_O_3_ mole fraction in the nanocomposite sensor, operating temperature, and acetone concentration) and dependent (sensor resistance ratio) variables have been introduced in Table 1.

**Table 1:** Acetone detecting ability of the In_2_O_3_/Fe_2_O_3_ nanocomposite sensors^4,8,9,21^.

Independent			Dependent
Range of the Fe_2_O_3_ content of nanocomposite (mole fraction)	Range of temperature (K)	Range of acetone concentration (ppm)	Range of SRR (-)
0–1	453–563	8.5–1000	1.13–21

**Figure 1:**
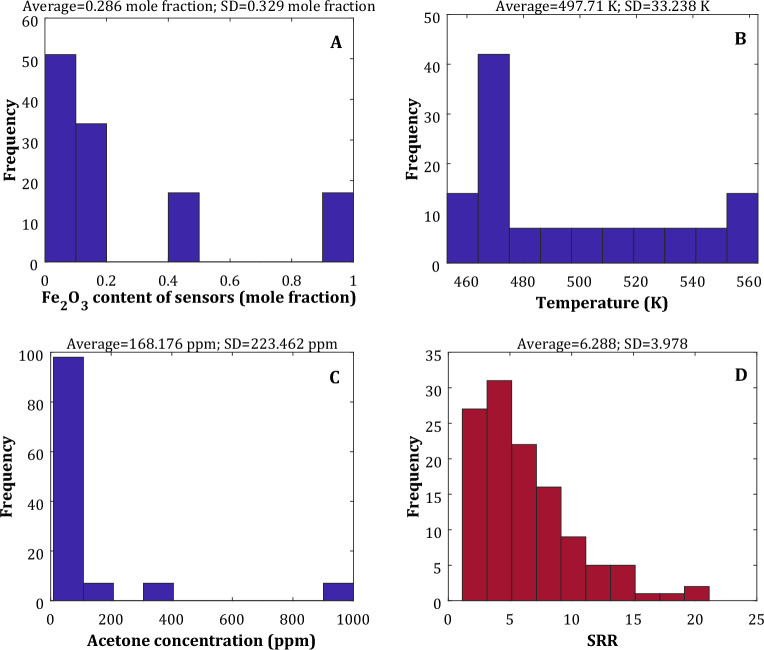
Histograms of the Fe_2_O_3_ content of nanocomposite sensors (**A**), operating temperature (**B**), acetone concentration (**C**), and SRR (**D**).

Figure 1 presents the histogram of independent and dependent variables gathered from the literature. This figure also reports the average and standard deviation (SD) of this collected database. Equations (1) and (2) have been utilized to calculate these statistical features^42^.

1 $$V^{ave} = \sum\limits_{j = 1}^{N} {V_{j} } /N$$

2 $$SD = \sqrt {\sum\limits_{j = 1}^{j} {\left( {V_{j} - V^{ave} } \right)^{2} /N} }$$

here, $V$ and $V^{ave}$ are the variable and its average value. The number of available datasets has been shown by *N*.

## Wavelet transform-artificial neural network

Both machine and deep learning methods are trustful tools to conduct sensitivity analysis^43^, parameter forecasting^44^, classification^45^, and control^46^ purposes. As the most popular machine learning approach, an artificial neural network (ANN) can be constructed by combining several processing nodes (i.e., neurons or nodes) in some interconnected successive neuronic layers^47^. The multi-layer perceptron (MLP) is a well-established ANN type that often includes two feedforward neuronic layers, namely hidden and output^48^. Since the number of output nodes equals the number of dependent variables, it is always known^49^. On the other hand, a suitable number of hidden nodes is often found by applying the trial-and-error technique.

The artificial neuron can be viewed as a combination of linear (L) and non-linear (NL) mathematical operations. The linear part (Eq. 3) combines the multiplication of the node’s entry vector (X) by the weight coefficients (W) and the bias (b).

3 $$L = \sum {WX + b}$$

The non-linear part is responsible for passing the linear part result through a specific equation, namely the transfer function (g). Equation (4) explains how the neuron’s output (out) has been achieved.

4 $$Out = g\left( {LP} \right)$$

Indeed, the transfer function helps the neuron and artificial neural network to simulate non-linear problems. The main limitation of the MLP model is that it can only be equipped with some pre-defined transfer functions^50^. The most widely-used transfer functions, like tangent and logarithm sigmoid and radial basis, are not non-linear enough to correlate highly non-linear problems precisely.

### Combining the MLP and wavelet transform

**Figure 2:**
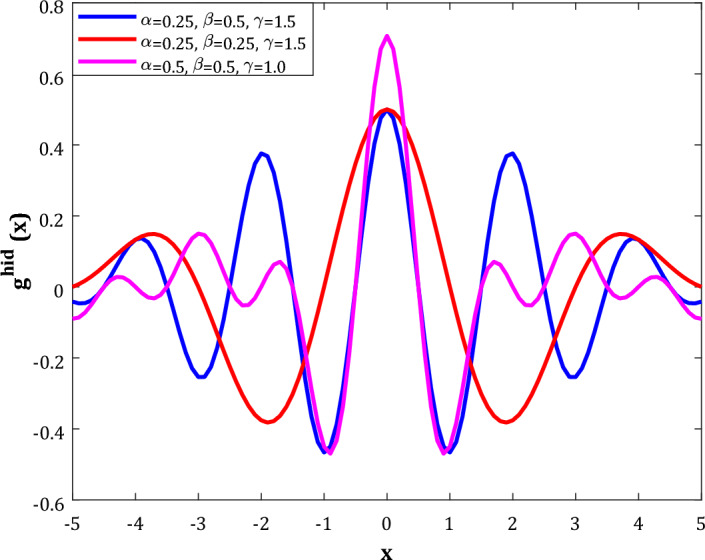
The general shape of the B-spline wavelet incorporated in the WT-ANN model.

Researchers have included the wavelet transforms in the MLP body as a transfer function to build the WT-ANN model^51,52^. The B-spline wavelet is a function with tunable nonlinearity often used as a transfer function in the WT-ANN’s hidden layer (*g*^*hid*^)^53^. Equations (5) and (6) show the mathematical formula of the B-spline wavelet transfer function (BSWTF)^54^.

5 $$g^{hid} \left( x \right) = \sqrt a \phi \left( {ax/\gamma } \right)^{\gamma } \exp \left( {2\pi i\beta x} \right)$$

6 $$\phi \left( k \right) = \left\{ \begin{gathered} 1\quad \quad \quad \quad \quad \quad k = 0 \hfill \\ \sin \left( {\pi k} \right)/\pi k\quad k \ne 0 \hfill \\ \end{gathered} \right.$$

where α, β, and γ are the parameters related to the nonlinearity and shape of the BSWTF. The B-spline wavelet transfer functions with different α, β, and γ values have been depicted in Fig. 2. It can be observed that this function has enough nonlinearity to correlate even the most complex phenomena. Moreover, it is possible to engineer its shape by changing its shape-related parameters^55^.

### Estimating acetone detecting ability of the In_2_O_3_/Fe_2_O_3_ sensor

**Figure 3:**
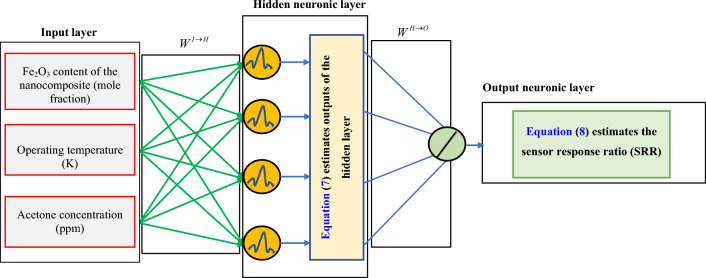
A typical WT-ANN with a 3-4-1 topology for estimating the acetone sensing ability of In_2_O_3_/Fe_2_O_3_ nanocomposites^56^.

Figure 3 presents the WT-ANN deployed to estimate the acetone-detecting ability of the In_2_O_3_/Fe_2_O_3_ sensor as a function of nanocomposite chemistry, operating temperature, and acetone concentration. It can be seen from Fig. 3 that the constructed WT-ANN constitutes an input layer and two neuronic layers of hidden and output. These layers are fully interconnected in a feedforward manner. The hidden layer has the B-spline wavelet transfer function, while the output layer equips with the linear transfer function.

This figure states that the vector of independent variables (*X*) is fully connected to the hidden layer’s nodes by weighted links ($W^{I \to H}$). Some mathematical processes based on Eq. (7) have been imposed on the entry vector of X to the hidden layer to achieve the outlet vector ($HL^{out}$).

7 $$Out^{HL} = g^{hid} \left\{ {\left( {\sum {W^{I \to H} \times X - b} } \right)/a} \right\}$$

The adjustable coefficients between the input and hidden layers are $W^{I \to H}$, a, and b. Equation (7) approves that the B-spline wavelet transfer function has been incorporated in the hidden layer of the WT-ANN.

Since the WT-ANN’s output layer has the linear transfer function, it is possible to achieve the predicted sensor resistance ratio (*SRR*^*pred*^) by multiplying the *Out*^*HL*^ and the weighted connections between the output and hidden layers ($W^{H \to O}$).

8 $$SRR^{pred} = \sum {W^{H \to O} \times HL^{out} }$$

It should be noted that the $W^{H \to O}$ shows the adjustable coefficients between the output and hidden layers.

All WT-ANN coefficients have been tuned during the cross-validation step employing an appropriate optimization algorithm^57,58^. The literature has extensively explained the tuning procedure of the $W^{I \to H}$, $W^{H \to O}$, a, and b^51^.

## Results and discussions

### Determining the best structure of the WT-ANN

The BSWTF parameters^53^ and the number of hidden nodes^51^ are those structural properties of the WT-ANN that should be selected appropriately. This study uses the genetic algorithm^59^ and the trial-and-error techniques to find these two structural properties, respectively. The deviation between actual and predicted SRRs is an objective function that must be minimized to construct the WT-ANN appropriately. The root mean squared error (RMSE), absolute average relative deviation (AARD%), MAE (mean absolute error), and coefficient of regression (R^2^) measure this deviation. Equations (9)–(12) express the formula of MAE, R^2^, MSE, and AARD%, respectively^60,61^.

9 $$MAE = \sum\nolimits_{j = 1}^{N} {\left| {SRR^{act} - SRR^{pred} } \right|} /N$$

10 $$R^{2} = 1 - \left( {\sum\nolimits_{j = 1}^{N} {\left( {SRR^{act} - SRR^{pred} } \right)_{j}^{2} }/\sum\nolimits_{j = 1}^{N}\left ( {SRR^{act} - SRR^{ave} } \right)_{j}^{2} } \right)$$

11 $$RMSE = \sqrt {\sum\nolimits_{j = 1}^{N} {\left( {SRR^{act} - SRR^{pred} } \right)_{j}^{2} /N} }$$

12 $$AARD\% = \sum\nolimits_{j = 1}^{N} {\left( {\left| {SRR^{act} - SRR^{pred} } \right|/SRR^{act} } \right)_{j} \times 100/N}$$

The actual measurements, WT-ANN predictions, and average values have been shown by the superscripts of *act*, *pred*, and *ave*, respectively.

### Simple flowchart of our study

**Figure 4:**
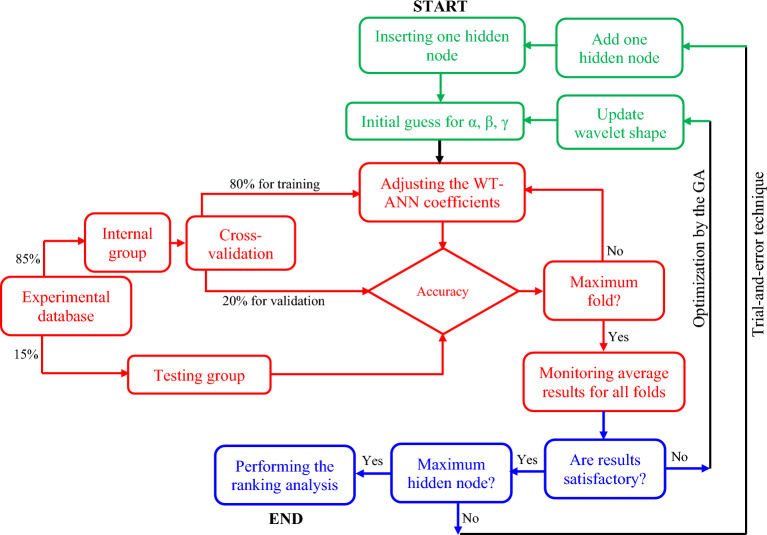
Constructing the WT-ANN model utilizing the trial-and-error technique and genetic algorithm (GA).

Figure 4 is an understandable flowchart to explain the processes followed to design the well-tuned WT-ANN model for estimating the acetone detecting ability of the In_2_O_3_/Fe_2_O_3_ sensors. This flowchart mainly includes three separate parts as follows:

I. Constructing the WT-ANN model (green part)
II. Five-fold cross-validation (red part)
III. Comparing the WT-ANNs performance to select a model with the highest prediction accuracy (blue part)

This flowchart also has two internal and external loops for adjusting the BSWTF parameters and WT-ANN coefficients, respectively.

### The best WT-ANN topology

It is obvious that at least one hidden node is required to build a WT-ANN with one hidden neuronic layer. Moreover, there is a rule of thumb to find the maximum allowable hidden nodes in the WT-ANN body^62^. The literature states that the numbers of the cross-validation datasets (here, 101 samples) should be at least two times the model’s tunable coefficients^62^. Therefore, the WT-ANN models may be built with a maximum of ten hidden nodes. Table 2 presents the highest accurate predictions obtained by different WT-ANN topologies differing with respect to the number of hidden nodes and BSWTF shape. The accuracy of the WT-ANN models over the cross-validation and testing groups and all database has been monitored by four statistical matrices. It can be concluded that the WT-ANN with α = β = 0.5 and γ = 1.0 and nine hidden nodes (highlighted by the gray color) is the highest precise model for estimating the acetone detecting ability of the In_2_O_3_/Fe_2_O_3_ nanocomposite sensors.

**Table 2:** The best results obtained by the different topologies of the wavelet neural network.

Number of hidden nodes	BSWTF adjusted parameters	Database	MAE	R^2^	RMSE	AARD%
1	α = 0.25	Cross-validation	2.1	0.57102	3.43	28.64
	β = 0.50	Testing	1.42	0.79069	2.18	25.86
	γ = 1.50	All samples	2	0.59666	3.27	28.22
2	α = 0.35	Cross-validation	1.94	0.61594	3.29	25.8
	β = 0.35	Testing	1.93	0.80285	2.63	27.73
	γ = 1.25	All samples	1.93	0.62769	3.2	26.09
3	α = 0.30	Cross-validation	1.24	0.86345	2.07	17.2
	β = 0.20	Testing	0.97	0.92351	1.36	17.83
	γ = 1.00	All samples	1.2	0.86869	1.98	17.29
4	α = 0.15	Cross-validation	0.98	0.91765	1.63	13.83
	β = 0.40	Testing	1.23	0.80449	1.83	18.66
	γ = 1.15	All samples	1.01	0.90767	1.67	14.56
5	α = 0.50	Cross-validation	1.03	0.9073	1.7	15.26
	β = 0.45	Testing	1.49	0.79078	2.22	22.5
	γ = 1.35	All samples	1.1	0.89266	1.79	16.35
6	α = 0.10	Cross-validation	1.01	0.92789	1.53	15.02
	β = 0.75	Testing	1.16	0.86314	1.66	18.07
	γ = 1.65	All samples	1.03	0.92094	1.55	15.48
7	α = 0.75	Cross-validation	0.8	0.95192	1.33	10.99
	β = 0.65	Testing	0.8	0.91012	1.2	12.4
	γ = 1.00	All samples	0.8	0.94828	1.31	11.2
8	α = 0.25	Cross-validation	0.73	0.95681	1.12	11.72
	β = 0.50	Testing	1.31	0.85726	2.5	15.41
	γ = 1.50	All samples	0.82	0.93552	1.41	12.28
9	α = 0.50	Cross-validation	0.7	0.95889	1.17	10.1
	β = 0.50	Testing	0.69	0.95184	0.95	10.18
	γ = 1.00	All samples	0.7	0.95813	1.14	10.12
10	α = 0.30	Cross-validation	0.8	0.92995	1.42	10.7
	β = 0.20	Testing	1.07	0.96622	1.57	17.1
	γ = 1.20	All samples	0.84	0.93518	1.44	11.67

**Figure 5:**
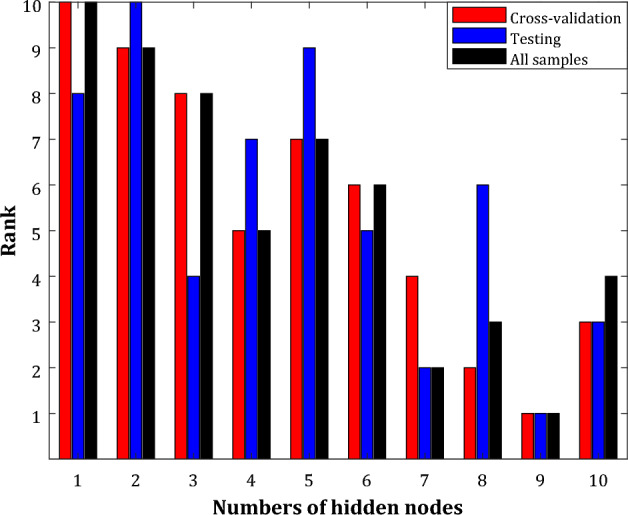
Rank order of different WT-ANN topologies differing from their number of hidden nodes and B-spline wavelet shape.

Although Table 2 shows the best topology of the wavelet transform-artificial neural network, it is better to use the ranking analysis to approve this matter further. The ranking places of different WT-ANN structures in the development and validation stages have been depicted in Fig. 5. Equation (13) has been used to calculate the average rank of each WT-ANN system over the RMSE, MAE, AARD%, and R^2^ indices.

13 $$Rank = round\left( {\sum\limits_{k = 1}^{4} {rank_{4} /4} } \right)$$

The ranking analysis also supports nine hidden nodes, and α = β = 0.5 and γ = 1.0 is the best WT-ANN topology for simulating the acetone-detecting ability of the In_2_O_3_/Fe_2_O_3_ nanocomposite sensors.

**Figure 6:**
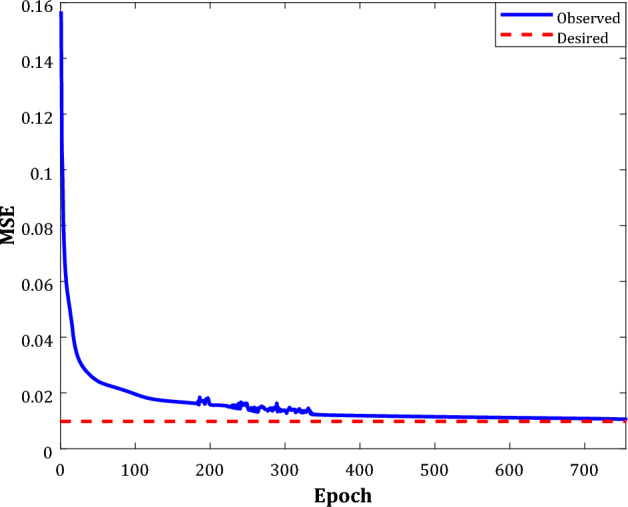
Decreasing the MSE by the learning algorithm in the cross-validation stage of the WT-ANN.

The learning algorithm’s performance to tune the coefficients of the optimum WT-ANN (i.e., W, a, and b) during the five-fold cross-validation has been illustrated in Fig. 6. This figure clarifies how the mean squared error (MSE) between the actual SRRs and their counterpart predictions by the WT-ANN continuously declines by increasing the number of optimizing tries (i.e., epoch). The observed MSE eventually reaches the desired value of 0.009 after ~ 750 epochs.

14 $$MSE = \sum\nolimits_{j = 1}^{N} {\left( {SRR^{act} - SRR^{pred} } \right)}_{j}^{2} /N$$

### Utilizing visual inspection to track the WT-ANN accuracy

**Figure 7:**
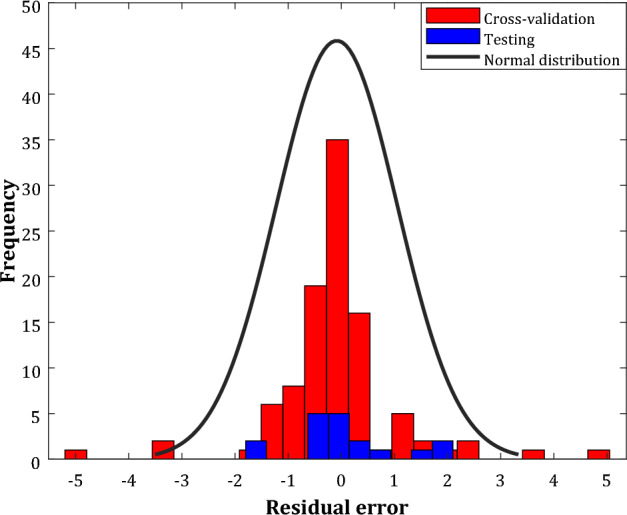
The histogram of the observed residual error by the optimum WT-ANN in the cross-validation and testing stages.

Figure 7 introduces the histogram of residual error (RE) between actual and estimated SRRs in the cross-validation and testing stages. The numerical values of this residual error can be obtained from Eq. (15).

15 $$RE = \left( {SRR^{act} - SRR^{pred} } \right)_{j} \quad j = 1,2, \ldots ,N$$

This figure approves an excellent performance of the optimum WT-ANN model for monitoring the acetone sensing by the In_2_O_3_/Fe_2_O_3_ nanocomposites. The built WT-ANN successfully predicts the sensor resistance ratio by the residual error ranges from -2 to 2.5. Moreover, this analysis states that 35 cross-validation and five testing samples have been estimated with a residual error equal to zero.

**Figure 8:**
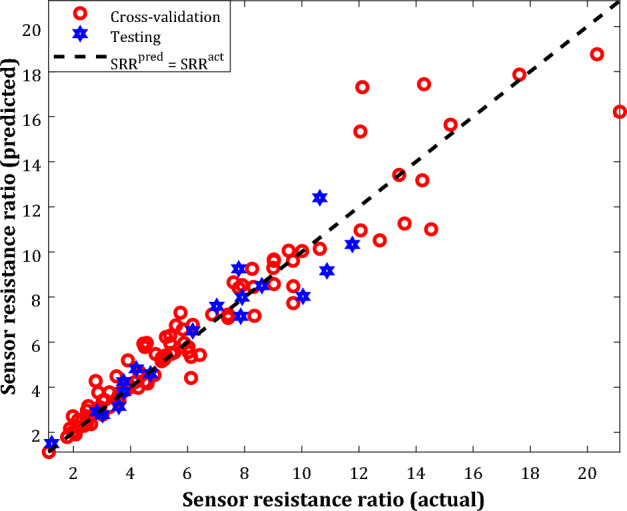
Correlation between predicted SRRs by the optimum WT-ANN and their related actual measurements.

The predicted SRRs by the well-structured WT-ANN model versus their laboratory-measured values in the cross-validation and testing stages have been separately indicated in Fig. 8. This figure states that the deployed WT-ANN model estimates the actual SRR values with acceptable accuracy. This finding can be highlighted as a confirmation of the excellent prediction capability of the proposed WT-ANN model. Excluding five testing samples that are not estimated well, all other instances have been well-correlated.

### Parametric study

**Figure 9:**
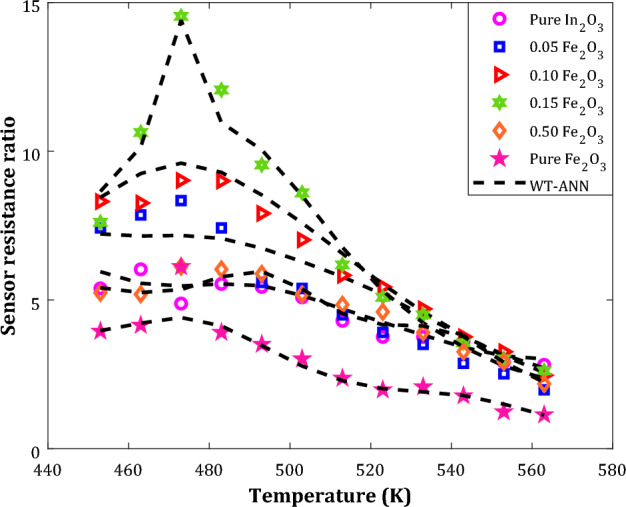
The influence of sensor chemistry and operating temperature on the acetone sensing (100 ppm).

The variation of the acetone detecting ability of nanocomposite sensors with different chemistry by temperature has been plotted in Fig. 9. This figure includes both experimentally reported samples and their counterpart estimations by the well-tuned WT-ANN model. A high level of agreement exists between the actual and predicted values of the sensor resistance ratios in a wide range of operating temperatures and nanocomposite chemistries.

Although pure Fe_2_O_3_ and In_2_O_3_ sensors have shown the minimum acetone detecting ability, their composites almost show better sensing performances. Generally, there is no specific trend for the acetone-detecting ability of nanocomposite with different chemistry. The acetone sensing ability of the In_2_O_3_-based nanocomposites increases by increasing their Fe_2_O_3_ molar content up to 15%, and after that, it decreases dramatically.

The In_2_O_3_-based nanocomposite sensor fabricated by 15 molar percent of Fe_2_O_3_ has the highest sensitivity for detecting the acetone agent in all temperature ranges. The 0.75In_2_O_3_/0.15Fe_2_O_3_ nanocomposite shows its maximum SRR at 473 K (200 °C). Despite this complex behavior, the constructed WT-ANN precisely identifies the SRR variation trend and estimates relatively all individual data samples.

**Figure 10:**
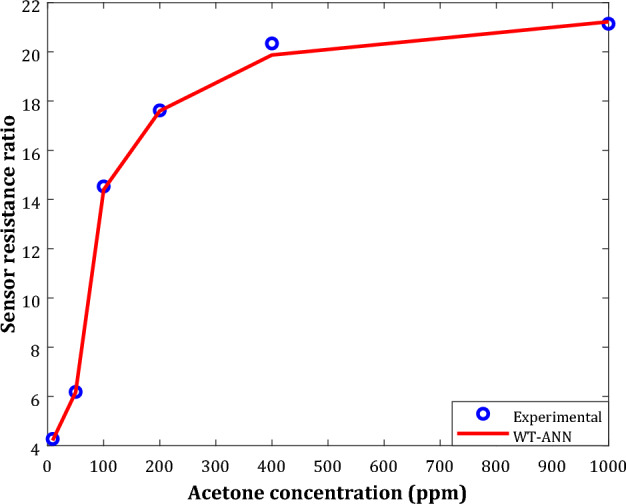
The effect of acetone concentration on the performance of the highest sensitive sensor (0.15 Fe_2_O_3_) at 473 K.

Experimental and modeling profiles of the SRR versus acetone concentration for the 0.75In_2_O_3_/0.15Fe_2_O_3_ nanocomposite sensor have been shown in Fig. 10. This figure confirms the high compatibility between actual and estimated SRR values.

It can also be concluded that the acetone-detecting ability of the given nanocomposite continuously improves by increasing the acetone concentration. Indeed, raising the acetone concentration from 10 to 1000 ppm increases the SRR by more than 400%.

## Conclusions

A straightforward intelligent correlation based on the wavelet transform-artificial neural network has been built to calculate In_2_O_3_/Fe_2_O_3_ sensor resistance ratio in the air with respect to the acetone from nanocomposite chemistry, temperature, and acetone concentration. Combining the genetic algorithm and trial-and-error analysis approved that a WT-ANN model with only nine hidden neurons and α = β = 0.5 and γ = 1.0 is the highest accurate model for the considered task. The deployed WT-ANN shows an incredible performance for precisely estimating 119 SRR data points of the nanocomposites ranging from pure In_2_O_3_ to pure Fe_2_O_3_. The overall MAE = 0.7, AARD% = 10.12%, RMSE = 1.14, and R^2^ = 0.95813 have been presented by the WT-ANN for calculating the SRR at wide ranges of acetone concentration, sensor chemistry, and temperature. The modeling results indicated that 0.75In_2_O_3_/0.15Fe_2_O_3_ nanocomposite has the highest acetone sensing ability over wide ranges of operating conditions. The proposed WT-ANN model in this study can help full-automating the acetone detecting ability of the In_2_O_3_/Fe_2_O_3_ sensor and enhance the knowledge about the sensor behavior in different operating conditions.

## Data Availability

All collected data from the literature - that are analyzed in this study - are available on reasonable request from the corresponding author.
