# Author Correction: Wavelet-artificial neural network to predict the acetone sensing by indium oxide/iron oxide nanocomposites

**DOI:** 10.1038/s41598-023-46411-6

**Published:** 2023-11-07

**Authors:** Reza Iranmanesh, Afham Pourahmad, Danial Soltani Shabestani, Seyed Sajjad Jazayeri, Hamed Sadeqi, Javid Akhavan, Abdelouahed Tounsi

**Affiliations:** 1 Faculty of Civil Engineering, K.N. Toosi University of Technology, No. 1346, Vali Asr Street, Mirdamad Intersection, Tehran, Iran; 2 Department of Polymer Engineering, Amirkabir University of Technology, Tehran, 1591634311 Iran; 3 Department of Chemistry, Mashhad Branch, Islamic Azad University, Mashhad, Iran; 4 Department of Chemical Engineering, Abadan Azad University, Khuzestan, Iran; 5 Department of Internet and Wide Network, Iran Industrial Training Center Branch, University of Applied Science and Technology, Tehran, Iran; 6 Mechanical Engineering Department, Stevens Institute of Technology, 1 Castle Point Terrace, Hoboken, NJ 07030 USA; 7 Material and Hydrology Laboratory, Civil Engineering Department, Faculty of Technology, University of Sidi Bel Abbes, Sidi Bel Abbès, Algeria; 8 Department of Civil and Environmental Engineering, King Fahd University of Petroleum & Minerals, Dhahran, 31261 Eastern Province Saudi Arabia

Correction to: *Scientific Reports* 10.1038/s41598-023-29898-x, published online 14 March 2023

The original version of this Article omitted an affiliation for Abdelouahed Tounsi. The correct affiliations are listed below.

Material and Hydrology Laboratory, Civil Engineering Department, Faculty of Technology, University of Sidi Bel Abbes, Sidi Bel Abbès, Algeria.

Department of Civil and Environmental Engineering, King Fahd University of Petroleum & Minerals, Dhahran, 31261, Eastern Province, Saudi Arabia.

The original Article has been corrected.

